# Electroacupuncture Relieves CCI-Induced Neuropathic Pain Involving Excitatory and Inhibitory Neurotransmitters

**DOI:** 10.1155/2019/6784735

**Published:** 2019-10-20

**Authors:** Chun-Ping Huang, Yi-Wen Lin, Der-Yen Lee, Ching-Liang Hsieh

**Affiliations:** ^1^Chinese Medicine Research Center, China Medical University, Taichung 40402, Taiwan; ^2^Research Center for Chinese Medicine and Acupuncture, China Medical University, Taichung 40402, Taiwan; ^3^Graduate Institute of Acupuncture Science, College of Chinese Medicine, China Medical University, Taichung 40402, Taiwan; ^4^Graduate Institute of Integrated Medicine, College of Chinese Medicine, China Medical University, Taichung 40402, Taiwan; ^5^Department of Chinese Medicine, China Medical University Hospital, Taichung 40447, Taiwan

## Abstract

Neuropathic pain caused by peripheral tissue injuries to the higher brain regions still has no satisfactory therapy. Disruption of the balance of excitatory and inhibitory neurotransmitters is one of the underlying mechanisms that results in chronic neuropathic pain. Targeting neurotransmitters and related receptors may constitute a novel approach for treating neuropathic pain. We investigated the effects of electroacupuncture (EA) on chronic constriction injury- (CCI-) induced neuropathic pain. The mechanical allodynia and thermal hyperalgesia pain behaviors were relieved by 15 Hz EA but not by 2 and 50 Hz. These phenomena were associated with increasing *γ*-amino-butyric acid (GABA) receptors in the hippocampus and periaqueductal gray (PAG) but not N-methyl-D-aspartate receptors. Furthermore, excitatory neurotransmitter glutamate was decreased in the hippocampus and inhibitory neurotransmitter GABA was increased in the PAG under treatment with EA. These data provide novel evidence that EA modulates neurotransmitters and related receptors to reduce neuropathic pain in the higher brain regions. This suggests that EA may be a useful therapy option for treating neuropathic pain.

## 1. Introduction

Neuropathic pain is usually defined as pain caused by a lesion or dysfunction of the somatosensory nervous system, either peripherally or centrally, and is a notably complicated pain disorder. Peripheral tissue injuries induce a painful sensation that prompts the individual to protect the damaged region so it can heal. Several neuropathic pain symptoms originate from peripheral damage, such as chemotherapy-induced peripheral neuropathy, phantom limb pain, diabetic painful neuropathy, and carpal tunnel syndrome. Peripheral damage affects 7–10% of the general population [1].

Glutamate is one of the major excitatory neurotransmitters in the central nervous system, and one of its receptors—the N-methyl-D-aspartate (NMDA) receptor, which comprises a Ca^2+^-permeable ion channel—is more permeable to Ca^2+^ ions than others [2]. Activation of NMDA receptors induces an influx of Ca^2+^ and further activates Ca^2+^/calmodulin-dependent protein kinase 2 or protein kinases for signaling in the postsynaptic neuron [2]. NMDA receptor mediation of central sensitization is a major component of neuropathic pain in the spinal cord [3]. Notably, excitatory glutamatergic transmission in the higher brain regions, such as the hippocampus, plays a key role in the onset of chronic pain accompanied by comorbid affective, emotional, and cognitive disorders [4, 5]. *γ*-Amino-butyric acid (GABA) is one of the major inhibitory neurotransmitters and occurs mainly in the interneurons of the mammalian brain. GABA_A_ is a ligand-gated ionotropic receptor that is located mainly in postsynaptic neurons and initiates fast synaptic inhibition. Activation of the GABA_A_ receptor initiates the influx of Cl^−^ into the postsynaptic synapse to induce hyperpolarization and then increase the threshold of depolarization [6]. However, unbalanced neurotransmitters or neuromodulators mismatch painful sensory inputs to generate spontaneous painful sensations. Central sensitization disrupts the balance of glutamate and GABA distribution and further results in chronic neuropathic pain [7].

Acupuncture is widely administered for pain relief [8, 9] and is believed to modulate several neurotransmitters such as dopamine, glutamate, acetylcholine, GABA, and serotonin [10–12]. The Baihui (GV20) and Dazhui (GV14) acupoints both belong to the “Du meridian,” which directly communicates with the brain in accordance with traditional Chinese medicine theory. The GV20 acupoint is located on the highest point of the head where all the yang meridians meet and is used to treat neurological and psychiatric diseases such as stroke, depression, and anxiety. It exerts neurological and neuroprotective functions through phosphorylated cyclic AMP-response element-binding protein and brain-derived neurotrophic factor (BDNF) activation [13] and alpha7 nicotinic acetylcholine receptor-mediated anti-inflammation [14]. In addition, our previous studies have known that EA at GV20 and GV14 provides neuroprotection by the reduction of S100B-mediated neurotoxicity [15] and EA at Zusanli (ST36) can evoke excitatory signal in either peripheral or central levels [16]. EA that applies to Hua Tuo Jia Ji (paraspinal) can reduce CCI-induced neuropathic pain and also can increase the levels of GABA_A_ receptor in spinal cord [17]. However, the effect of acupuncture at GV20 and GV14 on neuropathic pain in the higher brain regions interacting with the brainstem modulation system is still unknown.

In the current study, we assessed the effect of EA on CCI-induced neuropathic pain. We determined that mechanical and thermal hyperalgesia were induced after neuropathic pain induction and further reversed by 15 Hz EA but not 2 and 50 Hz. These phenomena were associated with an increase of the GABA_A_ receptor by EA in the hippocampus and periaqueductal gray (PAG) but not NMDA receptors of rats. Moreover, the levels of glutamate were reduced in the hippocampus and the levels of GABA were raised in the PAG by EA.

## 2. Experimental Procedures

### 2.1. Subjects

Experiments were conducted using male Sprague-Dawley rats (*n* = 56) weighing 200–300 g purchased from BioLASCO Co. Ltd., Taipei, Taiwan. The rats were maintained under a 12/12 h light/dark cycle, and water and food were available ad libitum. The Animal Care and Use Committee of China Medical University approved the use of these animals, and all procedures were performed according to the Guide for the Use of Laboratory Animals (National Academy Press). The number of animals used and their distress were minimized.

### 2.2. CCI-Induced Neuropathic Pain Model

The rats were anesthetized with 3% isoflurane induction, and then the isoflurane that is changed to 2% and maintained during the right sciatic nerve was exposed. CCI was induced by ligating the nerve proximal to the trifurcation with four 4-0 chromic gut sutures. In the sham-treated control group (Con), the right sciatic nerve was exposed without ligation. The surgical site was closed immediately using a silk line, and then the rats were placed back in their cage as previously described [18]. This neuropathic pain model is different from the spinal nerve ligation (SNL) model as using a piece of 6-0 silk thread ligates the one (L5) or two (L5 and L6) segmental spinal nerves [19].

### 2.3. Acupuncture Manipulation

Acupuncture stimulation was delivered by using stainless steel needles (0.5 inch, 32 G, Yu Kuang, Taiwan) that were inserted into the muscle layer at a depth of 1 mm in the GV20 and Dazhui (GV14) acupoints under 2% isoflurane anesthetization. We applied 2, 15, and 50 Hz EA by delivering electrical stimulation with a Trio 300 electrical stimulator (Grand Medical Instrument Co. Ltd., Ito, Japan). The stimulator delivered 100 *μ*s square pulses of 1 mA for 20 min. In the neuropathic pain group, a needle was inserted into the GV20 and GV14 acupoints without electrostimulation.

### 2.4. Animal Behavior of Mechanical Allodynia and Thermal Hyperalgesia

Pain behaviors (each indicated group, *n* = 12) were examined on days 5–8 to ensure the establishment of neuropathic pain. Mechanical sensitivity was measured by testing the force of responses to stimulation with five applications of electronic von Frey filaments (IITC Life Sciences, CA, USA) after 30 minutes of EA or without EA. Withdrawal latency of the hind paw lifting up was measured to assess thermal hyperalgesia (hot) using a radiant heat test apparatus (IITC Life Sciences, CA, USA). The results are presented as the difference between the injured side (right) and control side (left) as previously described [18]. A cold plate test was used to assess thermal hyperalgesia (cold) during which the rats were placed on a cold plate apparatus (Panlab, Spain) at a temperature of 4°C. The total number of injured hind paw lifts was counted for 5 min.

### 2.5. Western Blot Analysis

The rats (each indicated group, *n* = 6) were anesthetized with 3% isoflurane and brains were immediately sectioned (−5.2 to −6.3 mm) from bregma for PAG. The left-side hippocampus and whole PAG were excised immediately to extract proteins as previously described [20]. In brief, the total proteins were prepared by homogenizing the tissues in a lysis buffer containing 50 mM Tris-HCl (pH 7.4), 250 mM NaCl, 1% NP-40, 5 mM EDTA, 50 mM NaF, 1 mM Na_3_VO_4_, 0.02% NaNO_3_, and 1× protease inhibitor cocktail. The extracted proteins (30 *μ*g per sample according to a bicinchoninic protein assay) were subjected to 8% SDS-Tris glycine gel electrophoresis and transferred to a polyvinylidene difluoride membrane. The membrane was blocked with 5% nonfat milk in a TBS-T buffer (10 mM Tris pH 7.5, 100 mM NaCl, and 0.1% Tween 20), incubated with the first antibody in TBS-T and 1% bovine serum albumin, and incubated for 1 h at room temperature. A peroxidase-conjugated anti-rabbit antibody (1 : 5000) was used as the secondary antibody. The bands were visualized using an enhanced chemiluminescencent substrate kit (Pierce) with LAS-3000 Fujifilm (Fuji Photo Film Co. Ltd.). If appropriate, the image intensities of specific bands were quantified with NIH ImageJ software (Bethesda, MD, USA). The protein ratios were obtained by dividing the target protein intensities by the intensity of *α*-tubulin in the same sample. The ratio was calculated and then adjusted by dividing the ratios from the same comparison group relative to the control group as described in our previous paper [16].

### 2.6. Immunohistochemical Staining

The rats (each indicated group, *n* = 6) were anesthetized with 3% isoflurane and then perfused transcardially with 4% paraformaldehyde. The paraffin-embedded sections were cut to a thickness of 15 *μ*m and pasted onto microslide glasses coated with APS. The sections were postfixed briefly with 4% paraformaldehyde for 3 min and then incubated with a blocking solution containing 3% BSA, 0.1% Triton X-100, and 0.02% sodium azide in PBS for 2 h at room temperature. After blocking, the sections were incubated at 4°C overnight with the primary antibodies prepared in blocking solution. The secondary antibody was goat anti-rabbit (1 : 500) antibody (Molecular Probes, Carlsbad, CA, USA). We incubated the slices with avidin-biotin horseradish peroxidase complex (1 h), washed them three times with 0.1 M Tris buffer (5 min each), and then developed them in diaminobenzidine tetrahydrochloride (1-2 min) before washing three times with 0.1 M Tris buffer (5 min each). Finally, the sections were incubated with 0.1 M Tris buffer to stop the reaction. The slides were mounted with cover slips, and then these slides were observed by using a CKX41 microscope with an Olympus U-RFLT50 power supply unit (Olympus, Tokyo, Japan) as described in our previous paper [16].

### 2.7. Metabolite Sample Preparation and Derivatization

The hippocampus and PAG (each indicated group, *n* = 6) were homogenized using 1.0 mm zirconium oxide bead grinding with a ratio of 1 mg of tissues to 10 *μ*L of ultrapure water and then centrifuged at 14,000 rpm for 10 min. Each supernatant was collected as an aliquot of 100 *μ*L and mixed with 300 *μ*L of 100% methanol. After 14,000 rpm centrifugation for 10 min, 150 *μ*L of supernatant from methanol extraction was transferred to a new microtube and then subjected to vacuum drying. Each dry metabolite sample was combined with 16 *μ*L of ultrapure water, 2 *μ*L of 0.5 M carbonate buffer, pH 9.4, and then 2 *μ*L of 10 mg/mL dansyl chloride prepared in acetone. The reaction was conducted at 60°C for 2 h and then 100 *μ*L of ultrapure water was added for another 30 min incubation at 60°C. After 14,000 rpm centrifugation for 10 min, the supernatant of the dansylated sample was transferred to an insert vial and kept in an autosampler at 10°C for further analysis.

### 2.8. Liquid Chromatography-Electrospray Ionization-Tandem Mass Spectrometry (LC-ESI-MS)

The LC-ESI-MS system consisted of an ultraperformance liquid chromatography (UPLC) system (ACQUITY UPLC I-Class, Waters) and an ESI/APCI source of 4 kDa quadrupole time-of-flight mass spectrometer (Waters VION, Waters). The flow rate was set at 0.2 mL/min with a column temperature of 35°C. Separation was performed with reversed-phase LC on a BEH C18 column (2.1 × 100 mm, Walters) with 5 *μ*L sample injection. The elution started from 99% mobile phase A (ultrapure water + 0.1% formic acid) and 99% mobile phase B (100% methanol + 0.1% formic acid), held at 1% B for 0.5 min, raised to 90% B at 5.5 min, held at 90% B for 1 min, and then lowered to 1% B at 1 min. The column was equilibrated by pumping 1% B for 4 min. An LC–ESI–MS chromatogram was acquired using ESI+ mode under the following conditions: capillary voltage of 2.5 kV, source temperature of 100°C, desolvation temperature at 250°C, cone gas maintained at 10 L/h, desolvation gas maintained at 600 L/h, and acquisition by MSE mode with a range of 100–1000 m/z and 0.5 s scan time. The acquired data were processed using UNIFI software (Waters) with an illustrated chromatogram and summarized in an integrated area of signals. The results are presented as a ratio compared with normal control rats.

### 2.9. Statistical Analysis

All the data are expressed as the mean ± standard error. Significant differences were analyzed using one-way analysis of variance, followed by Tukey's post hoc test. A *p* value less than 0.05 was considered statistically significant.

## 3. Results

### 3.1. Electroacupuncture Reduced CCI-Induced Neuropathic Pain Behaviors

**Figure 1:**
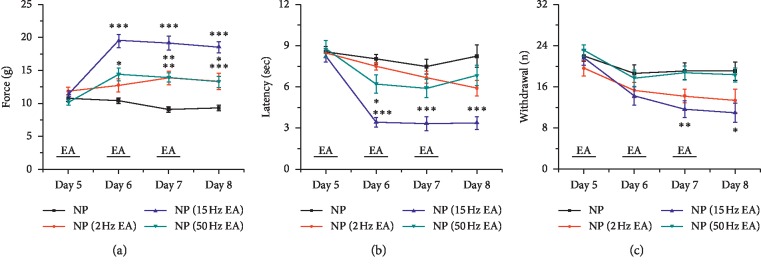
EA at GV20 reduced CCI-induced mechanical and thermal pain behaviors. Animal pain behaviors were tested at days 5–8 after CCI injury (*n* = 12). (a) Mechanical allodynia was measured by electronic von Frey filaments (g); (b) thermal hyperalgesia (hot) was measured by radiant heat and withdrawal latency (s) is presented as the difference between injured and control sides; (c) thermal hyperalgesia (cold) was measured using a cold plate apparatus and the number of injured hind paw lifts was counted for 5 min (*n*). NP, neuropathic pain; EA, electroacupuncture. ^*∗*^*p* < 0.05, ^*∗∗*^*p* < 0.01, and ^*∗∗∗*^*p* < 0.001 compared with the NP group.

As a first step to examine the effect of EA on neuropathic pain, we investigated 2, 15, and 50 Hz EA in a CCI neuropathic pain model. A typical mechanical allodynia was induced from days 5–8 after CCI induction and reduced using 2, 15, and 50 Hz EA (Figure 1(a), ^*∗*^*p* < 0.05, ^*∗∗*^*p* < 0.01, ^*∗∗∗*^*p* < 0.001 compared with the neuropathic pain group; NP). Next, we tested whether EA would also alter the thermal pain threshold. The radiant heat test indicated a significant decrease in paw withdrawal latency using 15 Hz EA (Figure 1(b)). By using the cold plate, we further determined that thermal hyperalgesia could only be reversed by 15 Hz EA rather than 2 or 50 Hz (Figure 1(c)).

### 3.2. GABA_A_ Receptor Increases from EA in the Hippocampus

**Figure 2:**
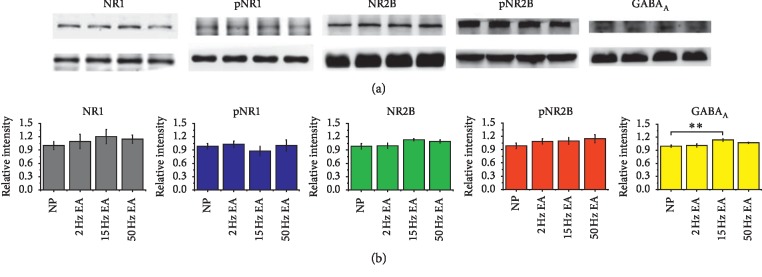
Expression of NR1, pNR1, NR2B, pNR2B, and GABA_A_ in the hippocampus. (a) The expression levels in tissues from the NP and 2, 15, and 50 Hz EA groups (from left to right; *n* = 6). The western blot bands at the top illustrate the target protein and the lower bands are internal controls (*α*-tubulin). (b) Densitometry analysis of protein level was quantified with NIH ImageJ software. Relative intensity was calculated as ratio of the NP group. ^*∗∗*^*p* < 0.01 compared with the NP group.

**Figure 3:**
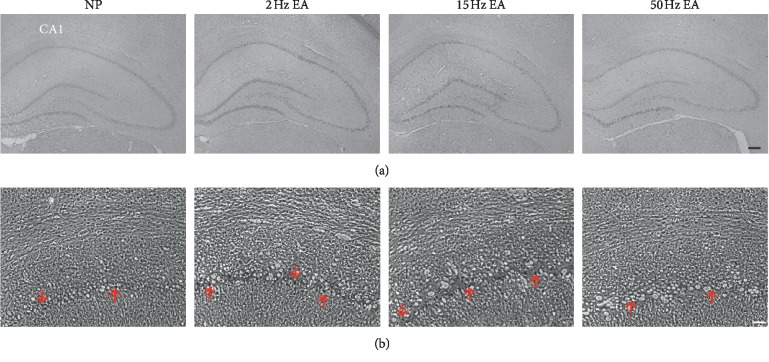
Example of GABA_A_ expression in the hippocampus. (a) Expression of GABA_A_ receptor was tested by immunohistochemical staining in the hippocampus (scale bar = 200 *μ*m); (b) arrows indicate the distribution of GABA_A_ in the CA1 region of hippocampus (scale bar = 50 *μ*m).

NMDA and GABA are the main excitatory and inhibitory neurotransmitters in the peripheral and central nerve systems that account for neuropathic pain. The three major NDMA receptors NR1, NR2, and NR3, which are distributed in the peripheral and central nervous system [21, 22], and the GABA_A_ receptor are major targets for sedative effects [23, 24]. Our data indicated that EA had no effects on the NR1, NR2B, or phosphorylated NMDA receptors or the pNR1 and pNR2B receptors (Figure 2(a)) in the rats' hippocampi, which respond to the ascending pain pathway. In addition, the level of the GABA_A_ receptor increased in response to 15 Hz EA treatment (Figure 2(b), ^*∗∗*^*p* < 0.01, compared with the NP group), which corresponded to behavioral observations, as evaluated using Western blot analysis. We further examined expression of the GABA_A_ receptor using immunohistochemical staining in the hippocampus (Figure 3(a)). The results indicated that the hippocampus demonstrated numerous distributions of GABA_A_ receptors, especially in the CA1 region (Figure 3(b)).

### 3.3. GABA_A_ Receptor Is Increased by EA in the PAG

**Figure 4:**
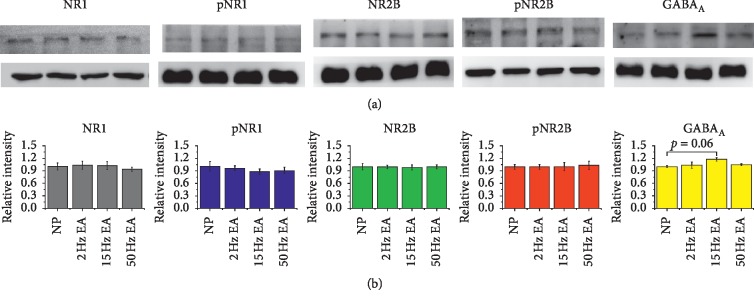
Expression of NR1, pNR1, NR2B, pNR2B, and GABA_A_ in the PAG. (a) The expression levels in tissues from the NP and 2, 15, and 50 Hz EA groups (from left to right; *n* = 6) in the aforementioned description. (b) 15 Hz EA increased the level of GABA_A_ receptor (*p*=0.06 compared with the NP group). Relative intensity was calculated as a ratio of the NP group.

**Figure 5:**
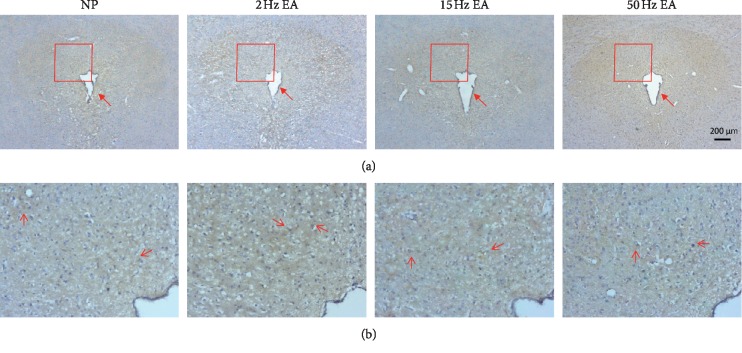
GABA_A_ receptors expressed in the PAG. (a) The immunohistochemical staining results of the GABA_A_ receptor are shown in the PAG (scale bar = 200 *μ*m). Arrow in the (a) indicates aqueduct; (b) arrows in the (b) indicate the distribution of GABA_A_ in the dorsolateral PAG.

To further test if EA can regulate the descending pain pathway, we investigated the aforementioned molecules in the PAG area. Our data revealed that 15 Hz EA increased the level of GABA_A_ receptor (Figure 4(b), *p*=0.06, compared with the NP group) but had no effects on NR1 and NR2B or pNR1 and pNR2B, corresponding to hippocampal observation. The results of immunohistochemical staining in the PAG also demonstrated considerable GABA_A_ receptor expression (Figure 5(a)), particularly in the dorsolateral PAG (Figure 5(b)) zone as indicated (red).

### 3.4. Electroacupuncture Reduces Glutamate in the Hippocampus and Increases GABA in the PAG

**Figure 6:**
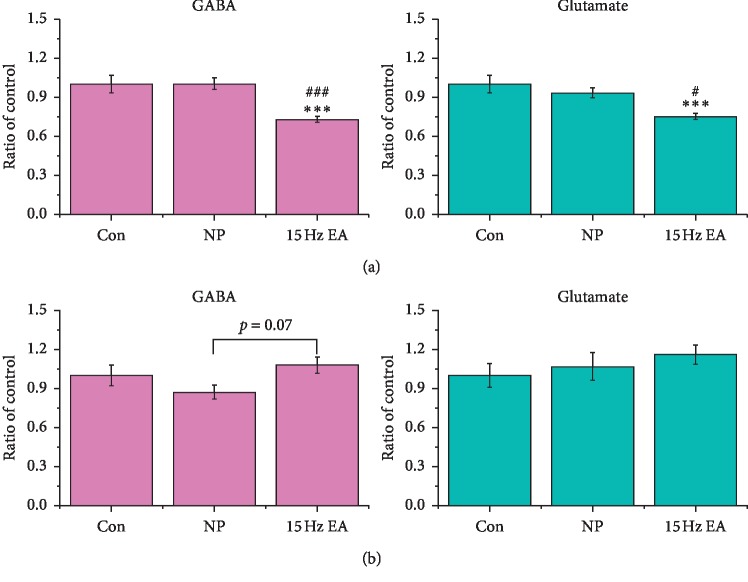
Neurotransmitters of glutamate and GABA were measured by the LC-ESI-MS system. (a) Samples of hippocampus from the control, NP, and 15 Hz EA groups were graphed as a ratio of the Con group (*n* = 6; ^*∗∗∗*^*p* < 0.001 compared with the Con group; ^#^*p* < 0.05, ^###^*p* < 0.001 compared with the NP group); (b) samples of PAG from the control, NP, and 15 Hz EA groups were graphed as a ratio of the Con group (*n* = 6). Fifteen-hertz EA increased GABA compared with the NP group (*p*=0.07).

To estimate the neurotransmitter changes induced by 15 Hz EA, lysates of rat hippocampus and PAG were prepared into dansylated samples and further measured by the LC-ESI-MS system. Our data indicated that 15 Hz EA significantly reduced the ratio of glutamate compared with the Con group (Figure 6(a), ^*∗∗∗*^*p* < 0.001) or NP group (Figure 6(a), ^###^*p* < 0.001) and also downregulated the ratio of GABA compared with the Con group (Figure 6(a), ^*∗∗∗*^*p* < 0.001) or NP group (Figure 6(a), ^#^*p* < 0.05) in the hippocampus. Noticeably, 15 Hz EA increased the ratio of GABA compared with the NP group but had no effects on glutamate in the PAG. However, this phenomenon had no significant difference compared with the NP group (Figure 6(b), *p*=0.07).

## 4. Discussion

These data provide the first animal experimental evidence that EA modulates excitatory and inhibitory neurotransmitters to relieve neuropathic pain in the higher brain regions. The hippocampus plays an integral role in the transition from acute to chronic pain in the limbic system [5, 25] and is involved in the processing and modification of nociception [26, 27]. It receives nociceptive inputs from ascending pathways and further prolongates subsequent nociceptive stimuli to activate descending pathways [28]. Blocking NMDA receptors in the hippocampal CA1 region alleviates nociceptive behaviors and processing related to persistent pain [29]. After 3 days of 15 Hz EA at GV20 and GV14, our data indicated that glutamate was reduced in the hippocampus (Figure 6(a)). We also observed that GABA was inhibited by EA. This phenomenon may lower the expression of BDNF and further modulate neuronal plasticity, which suggests that treatment with EA could prevent the development of chronic pain. EA at the Zusanli (ST36) and Sanyinjiao (SP6) acupoints can reduce pain sensation by decreasing NMDA receptor activation and this phenomenon can also be obtained by injecting dizocilpine, an NMDA receptor antagonist [30]. He and colleagues suggested that the threshold of long-term potentiation (LTP) from the C-fiber in the spinal dorsal horn was lower in neuropathic rats. The amplitude of the field potentials was higher in neuropathic pain rats. Notably, EA at ST36 and SP6 can reliably reduce neuropathic pain by inducing long-term depression (LTD) in the C-fiber [31]. The curative effect can be further reversed by MK-801 (an NMDA receptor antagonist) and naloxone (an opioid receptor antagonist). Similar to LTP in CNS, synaptic potentiation is formed by potentiating the NMDA subtype of the glutamate receptor.

Spontaneous mechanical or thermal hyperalgesia has often been observed with neuropathic pain. Neuropathic pain results from spontaneous or evoked pain, which is a common cause of chronic pain with unsatisfactory therapy. Opiates and anticonvulsive medicines are often used to treat neuropathic pain with nonspecific, insufficient, and life-threatening side effects including nausea, sedation, constipation, and tolerance [32]. Central sensitization of nociceptive synaptic transmission may be initiated from inflammatory, neuropathic, and postoperative pain syndromes. NMDA receptor activation may further induce Ca^2+^ influx to initiate second messenger pathways to deliver pain sensations. Intracellular Ca^2+^ is especially crucial in EA analgesia because it modulates spinal NMDAR [33]. NMDA receptors are long-term defined as nociceptive channels for inducing sensitization at the central spinal level. NMDA can be regulated by several protein kinases and phosphatases. Protein phosphatases 1 and 2A have been suggested to play crucial roles in EA analgesia by regulating the phosphorylation of NMDAR in the spinal cord [34]. Ryu et al. reported that the IB4 and NR1 double-labeled DRG neurons were increased after CFA-mediated inflammatory pain. The phenomenon can be further reversed by EA, suggesting possible mechanisms of NMDA receptors, especially in IB4-positive nociceptive neurons [34]. Furthermore, medial to high-frequency EA (10 and 100 Hz) reliably reduced CFA-initiated inflammatory pain. When EA is used simultaneously with a subeffective dose of MK-801, the antinociceptive effect is prolonged, which can provide a therapeutic method for clinical pain management [35]. Similar to these data, it has been documented that ketamine, an NMDA receptor antagonist, can enhance the antinociceptive effect of EA at low and high frequencies (2 and 100 Hz) in rats suffering neuropathic pain [36].

Pharmacologic inhibition of intrinsic GABA tone can cause tactile allodynia and thermal hyperalgesia in normal rats, whereas exogenous administration of GABA agonists can reverse allodynia and hyperalgesia in SNL-induced neuropathic pain rat model [37]. Spinal cord injury can cause neuronal hyperexcitability and glial activation, and these will disrupt the balance of chloride ions, glutamate, and GABA results in the generation of chronic neuropathic pain [38]. Therefore, GABA plays a critical role in neuropathic pain. Targeting selective benzodiazepine-sensitive GABA_A_ receptors is reported to be an alternative to opioids for treating chronic neuropathic pain [39]. An article indicated that selective activation of GABA_A_ receptors constituted an effective therapy for chronic neuropathic pain [40]. EA at low frequency can reduce pain that results from noradrenergic descending mechanisms involving spinal GABAergic modulation. By contrast, high-frequency EA at 100 Hz mainly acts on GABA_B_ mechanisms [41]. Low-frequency 2 Hz EA can reliably decrease pain signaling through GABA_A_ in the dorsal anterior pretectal nucleus (APtN). High-frequency EA at 100 Hz can reduce pain by *μ*-opioid and 5-HT1 receptors in the ventral APtN [42]. EA can reduce cold allodynia but not in the nonacupoint sham group. Injection of the GABA receptor antagonists gabazine or saclofen attenuated the therapeutic effect of EA on cold allodynia in rats. The aforementioned results indicate a crucial role for GABA_A_ and GABA_B_ receptors in tail neuropathic rats at spinal levels [43]. Low-frequency- and high-frequency-EA-induced analgesia may occur from activation of different receptors. EA at 2 Hz was curative for neuropathic pain with the expression of LTD in the C-fiber in SNL rats. The phenomenon can be blocked by NMDA and opioid receptor antagonists. By contrast, 100 Hz EA-induced LTP in SNL rats was mainly mediated by endogenous GABAergic and serotonergic inhibitory systems [44]. This suggests that neuronal hyperactivity in the spinal pain transmission was enhanced after nerve injury and further developed into neuropathic pain. Furthermore, EA may initiate the analgesic effect by increasing the expression of GABA and regulate GABAergic transmission on descending pathways in PAG [45]. Targeting these pathways might not only provide new therapies for pain but also prevent chronic pain following acute pain.

What is the biological significance of EA for relieving CCI-induced neuropathic pain? In the current study, our results suggested that 15 Hz EA at the GV20 and GV14 acupoints significantly reduced mechanical allodynia and thermal hyperalgesia. In addition, EA reliably increased the expression of inhibitory GABA_A_ receptors in the hippocampus and the level of GABA in the PAG. Furthermore, EA downregulated the excitatory glutamate neurotransmitter in the thalamus. Therefore, we support that these neurotransmitters had not nonspecific effects. Our findings provide crucial evidence of the efficacy of using EA in treating CCI-induced neuropathic pain and can be further translated to clinical practice.

One question needs to explain, why 50 Hz EA at GV20 and GV 14 can produce greater analgesic effect than these at the 2 Hz and 50 Hz EA. The spinal opioidergic, adrenergic, serotonergic, cholinergic, and GABAergic systems are involved in the analgesia mechanisms of EA on neuropathic pain. In addition, spinal *μ* and *δ* opioid receptors and GABAA and GABAB GABAergic receptors mediated via the descending inhibitory system in the CNS are also involved the analgesic effect of EA on neuropathic pain [46]. The EA at 2 Hz facilitates the release of *β*-endorphin, endomorphin, and enkephalin activating the *μ* and *δ* opioid receptors. The release of opioidergic substance and the activation of the receptors in EA at 15 Hz are similar to those EA at 2 Hz [47]. In addition, only 15 Hz EA could increase the levels of GABAA receptors in the thalamus. Therefore, 15 Hz EA produces greater analgesic effect on neuropathic pain than that at the 2 Hz and 50 Hz EA in this CCI-induced neuropathic pain model.

The limitation of the present study is that only six rats in each group are tested. If the number of the rats in each group increases, the *p* value of GABA receptor and the levels of GABA in the 15 Hz EA group compared with NP group in PAG may reach a significant difference of less than 0.05.

## Data Availability

The data used to support the findings of this study are available from the corresponding author upon request.
